# A Fairness-Enhanced Federated Learning Scheduling Mechanism for UAV-Assisted Emergency Communication

**DOI:** 10.3390/s24051599

**Published:** 2024-02-29

**Authors:** Chun Zhu, Ying Shi, Haitao Zhao, Keqi Chen, Tianyu Zhang, Chongyu Bao

**Affiliations:** 1 College of Science, Nanjing University of Posts and Telecommunications, Nanjing 210003, China; zhuchun@njupt.edu.cn; 2 School of Communication and Information Engineering, Nanjing University of Posts and Telecommunications, Nanjing 210003, China; 1022010215@njupt.edu.cn (Y.S.); b21011021@njupt.edu.cn (K.C.); b21011619@njupt.edu.cn (T.Z.); 3 College of Internet of Things, Nanjing University of Posts and Telecommunications, Nanjing 210003, China; 4 Portland Institute, Nanjing University of Posts and Telecommunications, Nanjing 210003, China; p21000108@njupt.edu.cn

**Keywords:** emergency communication, UAV-assisted communication, federated learning, client selection, fairness enhancement, multi-armed bandit (MAB)

## Abstract

As the frequency of natural disasters increases, the study of emergency communication becomes increasingly important. The use of federated learning (FL) in this scenario can facilitate communication collaboration between devices while protecting privacy, greatly improving system performance. Considering the complex geographic environment, the flexible mobility and large communication radius of unmanned aerial vehicles (UAVs) make them ideal auxiliary devices for wireless communication. Using the UAV as a mobile base station can better provide stable communication signals. However, the number of ground-based IoT terminals is large and closely distributed, so if all of them transmit data to the UAV, the UAV will not be able to take on all of the computation and communication tasks because of its limited energy. In addition, there is competition for spectrum resources among many terrestrial devices, and all devices transmitting data will bring about an extreme shortage of resources, which will lead to the degradation of model performance. This will bring indelible damage to the rescue of the disaster area and greatly threaten the life safety of the vulnerable and injured. Therefore, we use user scheduling to select some terrestrial devices to participate in the FL process. In order to avoid the resource waste generated by the terrestrial device resource prediction, we use the multi-armed bandit (MAB) algorithm for equipment evaluation. Considering the fairness issue of selection, we try to replace the single criterion with multiple criteria, using model freshness and energy consumption weighting as reward functions. The state of the art of our approach is demonstrated by simulations on the datasets.

## 1. Introduction

Numerous actors such as climate change, atmospheric circulation and human activities have led to a gradual increase in the frequency of extreme weather events, a phenomenon that can cause irreversible damage to communications infrastructure [1]. In such cases, emergency communication systems can provide alternate means of communication to compensate for the disruption of traditional communication systems [2].

The most cloud-based data communication and storage are in common form, in which case critical information in the affected area may be threatened by leakage [3]. The emergence of federated learning (FL) solves this problem to a certain extent. FL allows client devices to train on private datasets locally and only communicate and interact with the model. This training approach protects the data privacy of all parties and reduces the communication cost and network overhead [4]. In emergency communication scenarios, a large number of resources are required to meet emergency demands due to severe damage to the communication infrastructure [5]. However, there is competition for limited spectrum resources among different devices, which exacerbates the scarcity of spectrum resources. In this case, the training method using FL can also optimize the allocation and utilization of spectrum resources, thus improving the model performance of terrestrial devices.

At the same time, emergency communication often faces problems such as unfixed locations and complex and changing terrain. So, the flexibility of unmanned aerial vehicles (UAVs) can be utilized to reach remote areas [6] and assist terrestrial devices in deploying models. Terrestrial devices collect ground data in the affected area through sensors and cameras, etc., and local models can be obtained by processing these data to detect and categorize targets [7]. However, terrestrial devices with limited energy cannot store a large amount of data while repeating training to improve the accuracy of the model. UAV-assisted communication provides a feasible solution, where terrestrial devices upload the model parameters to the UAV. Furthermore, the UAV assumes the responsibility of aggregating the parameters, which allows the UAV to share part of the training energy consumption for terrestrial devices.

However, UAVs have limited energy, which is used not only to maintain their stay in a certain area [8] but also for their communication with terrestrial devices and computation of training models. In addition, the number of terrestrial Internet of Things (IoT) terminals is densely distributed, and the energy of the UAV cannot support it to receive model parameters from a large number of terrestrial devices and perform aggregation operations. Existing studies often use user scheduling to rationally optimize resource allocation, and we aim to use it to alleviate the energy consumption of the FL training. The UAV receives model parameters from only some of the terrestrial devices according to the scheduling strategy, fully utilizes the data resources of each device, and improves the overall learning effect and speed. In fact, due to the specificity of emergency communication scenarios, it is often difficult for us to predict the resource information of the terrestrial devices in advance, which will affect the reasonableness of the scheduling strategy. Therefore, in this paper, we adopt the method of multi-armed bandit (MAB) for client selection, and the MAB algorithm does not need to predict the actual resource situation of terrestrial devices [9].

We think further into this topic that the performance of local models may vary due to the heterogeneity of terrestrial devices in FL networks [10]. However, if we only focus on the energy consumption when making client selection, those terrestrial devices that consume slightly more energy may lose the opportunity to participate in the aggregation phase, so that the trained global model will have a poorer performance on the dataset of these devices, which will cause an unfairness problem. At the same time, the frequent transmission of data or frequent participation in model updates by some devices will increase the possibility of network congestion. All these factors will affect the real-time and stability of the emergency communication system, reduce the response speed and efficiency of the equipment, and even pose a threat to disaster relief and rescue. Consider this fairness issue, we introduce a model freshness metric in the reward function of the MAB algorithm, weighted with energy consumption, to replace a single criterion with multiple criteria.

More specifically, we summarize the main contributions of this paper below.

(1) Emergency communication scenarios are characterized by complex terrain, etc., and we use FL to optimize the allocation and utilization of spectrum resources so as to improve the model performance of terrestrial devices.
(2) The UAV energy consumption problem is mitigated by considering user scheduling strategies in the FL process and by selecting some devices to participate in the aggregation phase in order to achieve the goal of improving decision-making efficiency.
(3) In order to overcome the problem of it being difficult to predict the resource information of terrestrial devices, we adopt the MAB algorithm for client selection. Meanwhile, further considering the fairness of the scheduling strategy, we introduce model freshness, which is weighted with energy consumption and set as the reward function of the MAB algorithm.
(4) We conduct extensive simulation experiments on the MNIST dataset and incorporate fairness metrics to measure system performance.

The rest of the paper is organized as follows. Section 2 reviews the related literature. Section 3 describes the role of UAV-assisted communication in emergency communication scenarios and the FL process. We detail the MAB-based fair scheduling algorithm used to reduce energy consumption in Section 4. Section 5 describes the experimental evaluation. Finally, Section 6 summarizes this work.

## 2. Related Work

### 2.1. UAV-Assisted Communication

Currently, UAV-assisted communication is widely researched, and UAVs assume different roles in it. In the work of Hou et al. [11], in order to further improve the security of the communication, the UAV not only participates in the training process of FL but also acts as a jammer, transmitting noise interference to potential eavesdroppers. Study [12] also provides an anti-jamming framework for UAV-assisted networks that allows the UAV to jam eavesdroppers. Meanwhile, due to the long-distance transmission of communication data and the lack of reliability in communication between the local clients and the parameter server, the UAV can act as a relay node to assist in the training of the models. In [13], considering that different tasks in emergency communication scenarios have different optimization objectives, in the case of two types of services, UAVs are divided into two clusters based on the type of services, and different FL algorithms are applied to the two tasks so as to jointly optimize the cost of the two types of services. Under the architecture of hierarchical FL, Wang et al. [14] jointly optimize the key issues of matching UAVs with local devices, allocation of hierarchical FL time, and the number of local model iterations. In [15], the terminal device may offload a portion of the computation task to the UAV, which may choose to perform the computation or offload the bitstream to the access point again by matching. In addition, UAVs can be placed in the edge area close to the clients and can be used as edge servers to provide computation services. Wang et al. [16] utilize the UAV as a mobile base station to provide edge services, taking into account fairness, in ensuring user device shunt balancing and UAV load balancing. Considering the quality of service (QoS), study [17] uses the UAV to maximize the number of covered devices that satisfy different QoS requirements.

### 2.2. UAV-Assisted Communication under Energy Constraints

The flexibility of UAVs is due to their usually small size and weight, which results in limited on-board energy storage and their short time in the air. Therefore, there exists a growing body of work investigating UAV-assisted communication under energy constraints. Zeng et al. [18] investigate the energy efficiency of UAV point-to-point communication links by optimizing the UAV trajectory to strike a balance between maximizing the communication throughput and minimizing the energy consumption. In [19], a UAV ground secure communication system is designed by minimizing the secrecy rate under the constraints of maximum UAV transmit power, energy harvesting and artificial noise power level at the target node. Xiong et al. [20] in their work use the Markov decision process to describe the energy and data transmission optimization problem of the UAV as a way of maximizing the utility of the energy generated by the UAV. Ref. [21] proposes a novel system model with high energy efficiency that adapts to the network through UAV trajectory optimization. Considering the computational performance of the UAV and its limited energy, Jia et al. [22] upload the already encrypted model parameters to the safe zone of the UAV, where the UAV is only responsible for decryption and aggregation.

### 2.3. User Scheduling Policies for Energy Conservation

The key to user scheduling is how to select appropriate clients to participate in the FL process, and a reasonable selection of the client set can effectively alleviate the problem of energy scarcity. Zheng et al. [23] optimize the trade-off between energy consumption and system performance by selecting clients based on the learning time, data size and channel quality. In [24], client selection and energy management issues are jointly considered to optimize system performance based on channel state information, energy information and data quality. The work in [25] looks at significantly varying weight localization, performs user scheduling at each round, and selects clients containing important information for training, thus reducing the client energy cost while maintaining model accuracy. Han et al. [26] consider the energy consumption problem of training the same model repeatedly and design a client selection algorithm based on the energy-efficiency ratio in a FL framework. Considering the real-world environment with an uncertain resource situation, Ref. [27] uses trial and error based on a multi-armed bandit (MAB) algorithm to find a reasonable set of selection clients to improve the system performance.

### 2.4. Fairness Issues in Emergency Communication Scenarios

At present, the latest research on emergency communication is more inclined to achieve the goal of introducing infrastructure into the disaster-affected areas. Obviously, unbiased post-disaster reconstruction is extremely important, which is conducive to restoring life order and social stability and promoting economic recovery and development. Banerjee et al. [28] focus on the equitable distribution of communication opportunities among individual devices in their work, proposing an emergency communication system based on participation equity to ensure that all devices can benefit from collective action. Ho et al. [29] jointly optimize UAV height, power control, and bandwidth allocation with the goal of maximizing uplink throughput, considering the issue of link rate fairness. The work of Thantharate et al. [30] jointly optimizes the charging station assignment and UAV trajectories to optimize UAV fleet charging coordination, taking into account battery chemical constraints, flight time and charging station capacity. This method ensures a fair distribution of energy to maximize the drone’s flight time. Zhu et al. [31] convert the client selection problem into a MAB problem and utilize the techniques of upper confidence bound (UCB) policy and virtual queue to improve the availability of clients as well as the long-term fairness of the selection. Ref. [32] uses local loss values to accelerate convergence with fairness in mind, thereby improving the communication efficiency of the system. Different from these works, our work alleviates the energy consumption problem in the FL training process by adopting the user scheduling strategy and pays attention to the fairness of the opportunity for devices to participate in training, so as to improve the generalization of the global model.

## 3. System Model

### 3.1. Description of Emergency Communication Scenario

The use of UAV-assisted communication in emergency communication scenarios fundamentally optimizes the way we vulnerable casualty identification as well as disaster warnings. For example, in the case of bushfires, dry and hot weather can lead to fires. How to provide quick and timely warnings after a fire and how to accurately identify vulnerable casualties and provide rescue after a disaster are two issues that are critical to people’s lives.

**Figure 1:**
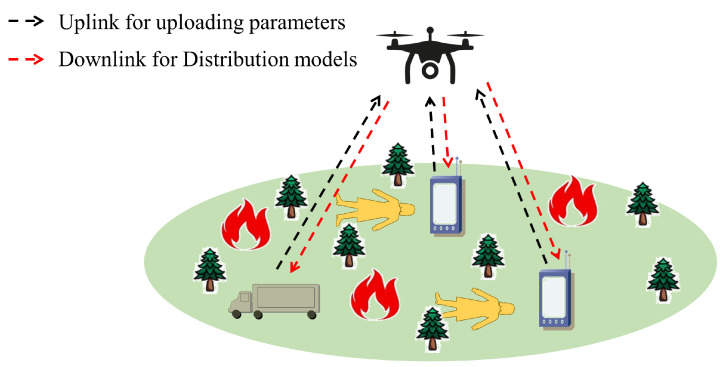
Data link description for terrestrial devices in emergency scenarios.

As shown in Figure 1, we consider intelligent vehicles, smartphones and other terminals as terrestrial devices, which collect images of the disaster area through sensors or cameras, and use the images of the disaster area to enrich the local private datasets while recognizing and predicting the targets. In order to improve the generalization of the models, the terrestrial devices need to interact with each other in a privacy-preserving manner. Deploying the UAV as a mobile base station can extend network coverage while improving wireless connectivity [33], which can better serve terrestrial devices. Rescuers can not only use the global model on the UAV to implement identification and classification of targets in the disaster area within the region but also use the ground terminal equipment to search along the way. The specific flow of this process is as follows.

(1) While training the local model using the local dataset, the terrestrial device sets up the communication settings through appropriate communication protocols to lay the foundation for establishing a reliable and smooth communication channel with the UAV.
(2) After the UAV receives the communication request from terrestrial devices, it carries out appropriate processing and establishes communication connections, and this process can use security measures such as authentication and encryption/decryption to ensure the confidentiality and integrity of the communications.
(3) After the UAV has performed the duties of the mobile base station, it distributes the fused parameter data to terrestrial devices. The terrestrial device receives the data and processes it accordingly to further improve the performance of the local model.

### 3.2. UAV-Assisted Federated Learning Model

**Figure 2:**
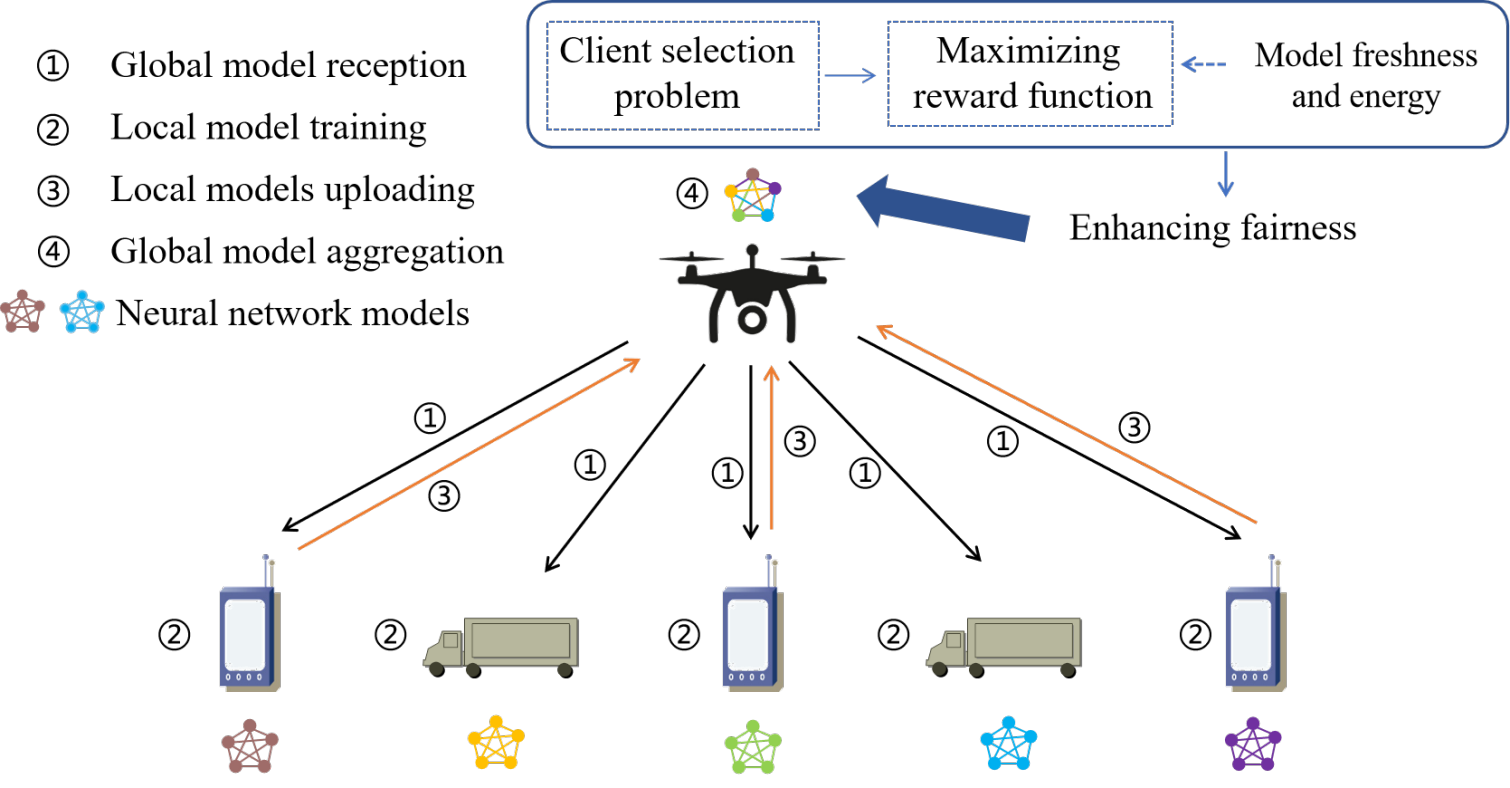
Data link description for terrestrial devices in emergency scenarios.

The task of the system is for multiple terrestrial devices to act as local training nodes and collaborate to train the global model using real-time disaster area data from the region. We consider a UAV-assisted FL model consisting of a UAV hovering in the air as well as a set of terrestrial devices with total number *M*, labeled using label $m=\left\{1,2,\cdots ,M\right\}$. For each terrestrial device $U_{m}$, it participates in the FL process in round $t=\left\{1,2,\cdots ,T\right\}$ as follows, which is shown in Figure 2.

(1) Global model reception: Terrestrial device $U_{m}$ receives the latest global model $w^{t-1}$ distributed by the UAV and uses it as the initial local model $w^{t-1}\to w_{m}^{t,0}$.

(2) Local model training: Terrestrial device $U_{m}$ performs a stochastic gradient descent (SGD) method to train a local model using a private dataset.

(1) $$w_{m}^{t,l}=w_{m}^{t,l-1}-\eta \nabla f_{m}\left(w_{m}^{t,l-1}\right)$$

where *l* is the number of local training rounds, ∇ is the gradient operation, η is the learning rate of the local model, and $f\left(w_{m}^{t,l}\right)$ is the loss function. After completing *l* rounds of local training, the model of terrestrial device $U_{m}$ can be denoted as $w_{m}^{t,l}\to w_{m}^{t}$.

(3) Local models uploading: Due to the limited energy of the UAV and terrestrial devices, it is not possible to continuously receive local models from all terrestrial devices. Therefore, the UAV selects some devices to participate in the aggregation according to the formulated policy, and the selected terrestrial devices upload the trained local model $w_{m}^{t}$ to the UAV. In this paper, orthogonal frequency division multiple access (OFDMA) is considered to achieve efficient utilization of spectrum resources and $n=\left\{1,2,\cdots ,N\right\},$ N<M is used to mark *N* sub-channels.

(4) Global model aggregation: The UAV takes the received local models and gets the latest global model $w^{t}$ of the *t*-th round through the average aggregation algorithm of FL.

(2) $$w^{t}=\frac{\sum_{m=1}^{N}D_{m}^{t}w_{m}^{t}}{\sum_{m=1}^{N}D_{m}^{t}}$$

where $D_{m}^{t}$ denotes the amount of data used by the terrestrial device $U_{m}$ for local training.

### 3.3. Energy Consumption Models

Our work focuses on the energy impact of user scheduling on the communication transmission, so we only focus on the uplink and downlink energy consumption of the UAV and terrestrial devices. Our work uses an OFDMA scheme with a bandwidth of *W* per sub-channel. The uplink energy consumption in round *t* is mainly the energy consumption of the terrestrial device $U_{m}$ to upload the local model, and we first calculate the uplink communication rate $r_{m,t}^{up}$ according to the following Equation (3).

(3) $$r_{m,t}^{up}=B\log_{2}\left(1+\frac{p_{m}{\left|g_{m,t}^{up}\right|}^{2}}{\sigma^{2}}\right)$$

where $p_{m}$ is the power of terrestrial device $U_{m}$, $g_{m,t}^{up}$ denotes the uplink channel gain of terrestrial device $U_{m}$ in round *t*, and σ is the noise power spectral density.

Then, the uplink time of terrestrial device $U_{m}$ is obtained as $\tau_{m,t}^{up}$.

(4) $$\tau_{m,t}^{up}=\frac{lo_{m,t}}{r_{m,t}^{up}}$$

where $lo_{m,t}$ denotes the size of the data volume of the local model parameter uploaded by the terrestrial device $U_{m}$.

Finally, we obtain the uplink energy consumption $E_{m,t}^{up}$ of the terrestrial device $U_{m}$ [34].

(5) $$E_{m,t}^{up}=p_{m}\tau_{m}^{up}$$

Similarly, the downlink energy consumption in round *t* is mainly the energy consumed by the terrestrial device $U_{m}$ to download the global model. We define the downlink communication rate of the terrestrial device $U_{m}$ as $r_{m,t}^{down}$.

(6) $$r_{m,t}^{down}=B\log_{2}\left(1+\frac{p_{m}{\left|g_{m,t}^{down}\right|}^{2}}{\sigma^{2}}\right)$$

where $g_{m,t}^{down}$ denotes the downlink channel gain between the UAV and the terrestrial device $U_{m}$ at round *t*. For simplicity, we assume that the transmit and receive powers of the terrestrial devices are numerically identical. The time at which the terrestrial device $U_{m}$ downloads the model is $\tau_{m,t}^{down}$.

(7) $$\tau_{m,t}^{down}=\frac{gl_{m,t}}{r_{m,t}^{down}}$$

where $gl_{m,t}$ denotes the data size of the global model parameters. Finally, the downlink energy consumption $E_{m,t}^{down}$ between the UAV and the terrestrial device $U_{m}$ can be obtained.

(8) $$E_{m,t}^{down}=p_{m}\tau_{m,t}^{down}$$

## 4. Design of User Scheduling Algorithms for Fairness Enhancement

In this subsection, we first formulate the problem and analyze it, then we introduce the proposed metrics to measure the extent to which the terrestrial device is out of the FL aggregation phase, and finally, we briefly describe our proposed algorithm under the MAB problem.

### 4.1. Problem Formulation and Analysis

Our goal is to propose an FL-based architecture that can be used to optimize energy consumption in a way that improves energy utilization and enhances system performance. Thus, we aim to minimize energy consumption while maintaining accuracy.

In round *t*, for terrestrial device $U_{m}$, the energy consumed up and down is $E_{m,t}$, whose value is the sum of the uplink energy and the downlink energy:

(9) $$E_{m,t}=E_{m,t}^{up}+E_{m,t}^{down}$$

We assume that the convergence condition $∥\nabla F\left(w^{T}\right)∥\leq \varepsilon ∥\nabla F\left(w^{T-1}\right)∥$ is reached after global iteration *t*, where $∥F∥$ is the parametric function, $F\left(w^{t}\right)$ denotes the global loss function in round *t*, and ε is the model accuracy with values ranging in $\left(0,1\right)$.

From this, we obtain the following optimization problem (*P*1):

(10) $$\begin{array}{cc} \left(P1\right) & \min\sum_{t=1}^{T}\sum_{m=1}^{M}Q_{T}\left(E_{m,t}\right)\\ s.t. & \left\{\begin{array}{cc} E_{m,t}=E_{m,t}^{up}+E_{m,t}^{down} & \left(C1\right)\\ \sum_{t=1}^{T}E_{m,t}\leq E_{m}^{\max} & \left(C2\right)\\ \sum_{t=1}^{T}E_{UAV,t}\leq E_{UAV}^{\max} & \left(C3\right)\\ m\in \left\{1,2,\ldots ,M\right\} & \left(C4\right) \end{array}\right. \end{array}$$

where $Q_{T}\left(E_{m,t}\right)$ is the energy required for the training round *T*, $E_{m}^{\max}$ is the maximum energy used by the terrestrial device $U_{m}$ for FL training, $E_{UAV,t}$ is the energy used by the UAV for FL in the *t* round, and $E_{UAV}^{\max}$ is the maximum energy used by the UAV for FL training. Constraint *C*1 reflects that the energy consumption related to terrestrial devices in round *t* consists of both uplink and downlink energies. Constraint *C*2 indicates that the energy consumed after round *T* cannot exceed the maximum energy used for FL by the terrestrial device $U_{m}$. Constraint *C*3 indicates that the total energy used by the UAV for FL is limited. Constraint *C*4 represents the total number of terrestrial devices involved in FL training.

We substitute Equations (5) and (8) into Equation (9) to change the energy of terrestrial device $U_{m}$ to $E_{m,t}=p_{m}\left(\tau_{m,t}^{up}+\tau_{m,t}^{down}\right)$. Furthermore, it is difficult to predict these two delays separately, which will greatly reduce the resource consumption, so we evaluate them as a whole in our work. As shown in *P*1 above, our work considers an energy-limited UAV as well as numerous energy-limited terrestrial devices. For the terrestrial devices, the limited energy cannot support them to upload their model parameters to the UAV for aggregation in each round of FL. While performing model aggregation requires the UAV to wait for all terrestrial devices to finish uploading their parameter data, the energy consumption of the UAV for hovering in the air during this time is also extremely high. So in our work, we set a time threshold $\tau^{max}$, which is the maximum time limit that allows the terrestrial devices to upload model parameters. We consider the use of a user scheduling policy in the aggregation phase of FL to select terrestrial devices with lower energy consumption, while keeping the latency of the whole process within a certain range. Thus, the problem transforms into the following (*P*2).

(11) $$\begin{array}{cc} \left(P2\right) & \min\sum_{t=1}^{T}\sum_{m\in S_{t}}^{N}p_{m}\tau_{m,t}\\ s.t. & \left\{\begin{array}{c} \left|S_{t}\right|=N<M\\ \tau_{m,t}=\tau_{m,t}^{up}+\tau_{m,t}^{down}\\ \tau_{m,t}\leq \tau^{\max}\\ S_{t}\subset \left\{1,2,\ldots ,M\right\} \end{array}\right. \end{array}$$

where $S_{t}$ is the set of selected terrestrial devices in round *t*, the total number of selected devices is the total number of sub-channels under the OFDMA scheme, and the value of *N* is less than the total number of terrestrial devices, taking into account resource constraints.

In order to avoid spending a lot of resources to predict the accurate resource information of all devices, we consider using the MAB algorithm to develop user scheduling policies. The MAB algorithm is a classical reinforcement learning algorithm that makes decisions by weighing exploration and utilization. For each round of training, different terrestrial devices are selected to explore the rewards that can be obtained, and previously well-performing terrestrial devices are utilized to obtain more rewards. We can convert the user scheduling problem of (*P*2) into a MAB problem, where the UAV minimizes energy consumption by maximizing the reward function through continuous trial and error. Therefore, formulating an appropriate reward function is the key to utilizing the MAB algorithm.

### 4.2. Design of a Measure of Model Freshness

We consider that model sharing may create additional fairness issues, and if only energy is used as a reward function, it may make certain terrestrial devices with higher energy consumption perform less well. At the same time, if certain terrestrial devices do not participate in the FL aggregation process for a long period of time, it can lead to too much deviation from the global model and too little accuracy on their local datasets, which is negative for optimizing the local model of the terrestrial devices.

We define a metric to measure the extent to which the terrestrial device participates in the FL aggregation process, aiming to weight it with energy consumption to balance the unfairness of a single criterion for device selection. We refer to this as model freshness, which is defined by the following Equation (12):

(12) $$FM_{m}^{t}=t-aC_{m}$$

where *a* is a constant with a general value of 1, and $C_{m}$ denotes the number of times terrestrial device $U_{m}$ has participated in the FL aggregation phase. We measure this by a counting function. If the terrestrial device $U_{m}$ has participated in round *t* then $C_{m}+1$, otherwise $C_{m}$ remains constant.

### 4.3. Proposed Fairness Algorithm Based on MAB Problem

If the terrestrial device $U_{m}$ has not been selected by the UAV to participate in the model aggregation phase of FL, it may lead to a gradual deviation of the global model from the local dataset of $U_{m}$. Furthermore, this deviation will bring about a decrease in the accuracy of models. In this paper, we define this as the backwardness of $w_{m}$, which can introduce information bias to the rescue of the injured and even pose a threat to their life and health. In energy-limited emergency communication scenarios, our algorithm takes into account the energy consumption while balancing the freshness of the model compared to partial work that only considers energy consumption. Our work replaces a single criterion with multiple criteria for user scheduling, which can lead to a fairer selection of each terrestrial device by the UAV. This fairness enhancement improves the generalization of the global model. The more generalized the global model is, the more beneficial it is for all terrestrial devices. Therefore, our approach can improve the performance of the whole system by reducing the energy consumption while ensuring the non-backwardness of the local models of terrestrial devices.

The MAB problem can be formally viewed as a time-series process. At each time step, the player chooses one of the arms to pull, and the system returns a reward associated with this arm. The player continually adjusts their decision making based on the rewards they receive in order to achieve the highest cumulative rewards. Thus, the key to the MAB problem is the trade-off between exploring unknown potential reward distributions and using the information available to maximize rewards. We assume that the UAV is the player, and terrestrial devices are the multiple arms of the multi-armed bandit. The player pulling an arm is the UAV selecting the terrestrial device $U_{m}$ to participate in the FL aggregation phase. Algorithm 1 demonstrates the FL framework and process for fairness enhancement, and we will explain user scheduling-related content in steps.

(1) Initialize: The distribution of the reward function for all terrestrial devices is unknown. This means that we do not know the probability of choosing which terrestrial devices to participate in the aggregation can get high rewards.

(2) Calculate the reward function: The reward function is the key in the MAB problem, which can be used to measure the contribution or effect of each device’s participation in the aggregation, so as to decide the resources or weights to be assigned to the devices. By rationally designing the reward function, the fairness and efficiency of resource allocation can be ensured, thus enhancing the overall effect of FL.

We normalize the energy of the terrestrial device $U_{m}$:

(13) $${\hat{E}}_{m,t}=1-\frac{E_{m,t}}{E_{t}^{\max}}\in \left[0,1\right)$$

where $E_{t}^{\max}$ is the maximum value of energy of terrestrial devices in round *t*. Then, we can get the average energy reward value as

(14) $${\bar{E}}_{m,t+1}=\frac{1}{C_{m}+1}\left[C_{m}{\bar{E}}_{m,t}+{\hat{E}}_{m,t}\right]$$

The model freshness introduced in the previous subsection is weighted with the average energy value to give the reward function as follows:

(15) $${\bar{\mu }}_{m,t}=\alpha {\bar{E}}_{m,t+1}+\left(1-\alpha \right)FM_{m}^{t}$$

where α is the equilibrium weight of energy versus model freshness with respect to the freshness of the model, which takes the range $\left[0,1\right]$.

(3) Update strategy: Our work uses a upper confidence bound (UCB) algorithm to rank the terrestrial devices for selection using UCB scores.

(16) $$UCB_{m,t}={\bar{\mu }}_{m,t}+\sqrt{\frac{2\log\left(\frac{1}{\xi }\right)}{C_{m}}}$$

where ξ is the hyperparameter, whose value here is $\xi =\frac{1}{t-1}$.

We sort the UCB scores of terrestrial devices in descending order, and the UAV selects the first *N* terrestrial devices to participate in the training of the aggregation stage. In other words, the selected set of devices is constructed according to the following rule.

(17) $$S_{t}=\underset{S_{t}}{\arg\max}\sum UCB_{m,t}$$

**Algorithm 1** A MAB-based fair scheduling algorithm for reducing energy consumption.
**Initialize:** global model $w^{0}$; *M* terrestrial devices indexed by *m*, each with a local data volume of $D_{m}^{t}$ in round *t*;**Output:** global model $w^{T}$  1:

TheUAV:

  2:Initialize global model $w^{0}$  3:**for** each round t=1 to *T* **do**  4:   $S_{t}$← ClientSelection(*t*, *N*),$\left|S_{t}\right|=N$  5:   Receive the trained local models $w_{m}^{t}$ from the terrestrial device in the set $S_{t}$  6:   Aggregate the models uploaded according to Equation (2)  7:   Send $w^{t}$ to *M* terrestrial devices  8:
**end for**
  9:

$\mathrm{The}\mathrm{terrestrial}\mathrm{device}U_{m}:$

10:Receive the global model $w^{t}$ from the UAV11:**for** each local round l=1 to *T* **do**12:  Train the local model $w_{m}^{t}$ according to Equation (1)13:
**end for**
14:Send $w_{m}^{t}$ to the UAV15:

ClientSelection(t,K,N):

16:Initialize the relevant parameters to ensure that the reward distribution is unknown17:Update the reward function according to Equation (15) and also update the UCB score according to Equation (16)18:Sort the UCB scores of individual terrestrial devices in descending order19:Select the first *N* terrestrial devices to form $S_{t}$, i.e., $S_{t}=\underset{S_{t}}{\arg\max}\sum UCB_{m,t}$20:Return $S_{t}$ to the UAV


## 5. Experimental Performance and Analysis

### 5.1. Simulation Environment and Dataset

(1) Simulation environment: We consider a FL network in the presence of a UAV and M=20 terrestrial devices that have an energy budget of 400 J and are randomly distributed over a radius of 200 m. We set the number of OFDMA subchannels N=10. Considering the heterogeneity of the terrestrial devices, we set the transmit power of the terrestrial devices to be uniformly distributed in $\left(p_{\min},p_{\max}\right)$, where the value of $p_{\min}$ is 0.1 W, and the value of $p_{\max}$ is 0.3 W. We use a Rayleigh distribution with uniform variance to represent small-scale fading, and use the path loss model $PL\left(\mathrm{dB}\right)=128.1+37.6\log_{10}\left(d\right)$ to describe large-scale fading, where $d\left(\mathrm{km}\right)$ denotes the distance. The large-scale fading and small-scale fading together form the channel gain. Considering the specificity of emergency communication scenarios, we set the number of local training rounds to 1 and the learning rate to 0.05. The specific parameters are shown in Table 1.

(2) Datasets: Image recognition in emergency communication scenarios is an extremely important area. The MNIST dataset we use has become one of the most representative datasets in the machine learning community and is widely used to test and compare the performance of different algorithms. In fact, in some specific cases, there is a need to confirm the identity information of the trapped person, which may involve the recognition and analysis of handwritten digits.

### 5.2. The Indicator of Fairness

Our work is concerned with fairness in user scheduling, so we need to use fairness metrics for evaluation. There are three common types of fairness metrics, which are listed below.

(1) Standard deviation std. For an arbitrary model *w*, the standard deviation is computed by testing the loss on *M* devices with the following formula:

(18) $$std\left(L_{m}{\left(w\right)}_{m\in \left[M\right]}\right)=\sqrt{\frac{\sum_{m=1}^{M}{\left(L_{m}\left(w\right)-\mu \right)}^{2}}{M}}$$

where $L_{m}\left(w\right)$ is the model’s test loss on the terrestrial device $U_{m}$ and $\mu =\frac{1}{M}\sum_{m=1}^{M}L_{m}\left(w\right)$ is the average of all test loss. A smaller standard deviation means a fairer distribution.

(2) Gini coefficient Gini. The Gini coefficient measures the degree of inequality in a distribution and is often used to measure the degree of inequality in the distribution of income, wealth, and so on. For any model $w_{1}$ and $w_{2}$, if $Gini\left(L_{m}{\left(w_{1}\right)}_{m\in \left[M\right]}\right)>Gini\left(L_{m}{\left(w_{2}\right)}_{m\in \left[M\right]}\right)$, then model $w_{1}$ is more equitable than model $w_{2}$.

(19) $$Gini\left(L_{m}{\left(w_{1}\right)}_{m\in \left[M\right]}\right)=\frac{\sum_{i=1}^{M}\sum_{j=1}^{M}\left|L_{i}\left(w_{2}\right)-L_{j}\left(w_{1}\right)\right|}{2M^{2}\nu }$$

where $\nu =\frac{1}{M}\sum_{m=1}^{M}L_{m}\left(w_{1}\right)$.

(3) Jain indicator. Jain is defined as follows:

(20) $$Jain=\frac{{\left(\sum_{m=1}^{M}acc_{m}\right)}^{2}}{M\times \sum_{m=1}^{M}ac{c_{m}}^{2}}$$

where $acc_{m}$ denotes the accuracy of the local model of the terrestrial device $U_{m}$. The Jain metric takes values in the range $\left(0,1\right)$. The closer the value is to 1, the more equitable the allocation of resources.

The standard deviation measures the deviation of individual device test losses from the mean, which is susceptible to very large or very small values in the array. Therefore, we believe that the deviation of the measurement from the mean does not present a good picture of the fairness performance of the system. The Jain metric is more widely used and easier to calculate than the Gini coefficient [35]. It is calculated using the accuracy of the models on the private datasets, which to some extent reflects the extent to which the global model contributes to the local models. The closer the value of the Jain metric is to 1, a direct indication is that there is little difference in the accuracy of models across terrestrial devices, which also indicates that the global model can be beneficial to all terrestrial devices for local training. This also indicates the fairer scheduling of terrestrial devices in the system.

### 5.3. Performance Comparison of Different Selection Criteria

We use two contrasting algorithms to highlight the sophistication of our algorithm.

(1) Gossip (stochastic greedy) selection [36]: The UAV adopts a randomized strategy for scheduling terrestrial devices, which is a traditional FL scheduling strategy full of randomness.
(2) Energy-oriented device selection [27]: The UAV evaluates terrestrial devices for scheduling with a single criterion, considering only the energy consumption and selecting only terrestrial devices with low energy consumption to participate in the aggregation phase.

**Figure 3:**
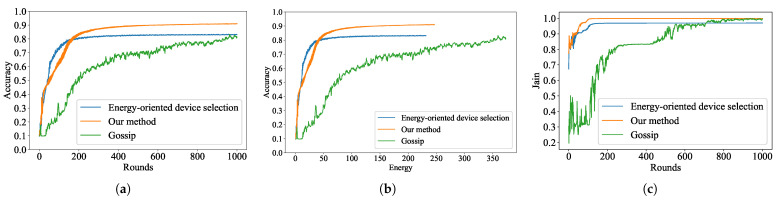
Comparisons of accuracy, energy and Jain for the three simulated user scheduling schemes on the MNIST dataset. (**a**) Testing accuracy versus global rounds *T*. (**b**) Testing accuracy versus total energy (J). (**c**) Comparison of global Jain metric.

After repeated simulation experiments, we present the final numerical results in Table 2. Figure 3a shows the accuracy of the three schemes on the MNIST dataset with the same number of global training rounds. It can be clearly observed that the accuracy of our scheme is about 91%, which is the best among the three schemes. This is because our scheme weights energy consumption with model freshness to balance the unfairness problem that arises when when the UAV performs user scheduling. This equality of opportunity optimizes the global model and also contributes to the performance of individual terrestrial devices. In contrast, the energy-oriented device selection scheme focuses only on energy consumption and may duplicate the selection of terrestrial devices with low energy consumption. The diversity of the model parameters involved in the aggregation decreases, which may lead to a decrease in model accuracy, as evidenced by the fact that the accuracy of the scheme in the simulation experiments is only 83%. Furthermore, the gossip scheme is full of randomness, which reduces the efficiency of the system’s training, as evidenced by the slower rate of convergence of the accuracy curves. Figure 3b shows the energy consumption of the three schemes with different accuracies on the MNIST dataset. It can be observed that energy-oriented device selection scheme has the lowest energy consumption, but this low energy consumption is obtained by sacrificing the accuracy. Combining accuracy and energy consumption, our scheme is the best.

We evaluate the fairness of user scheduling using the Jain metric and display the results in Figure 3c. It is obvious that in our scheme, the Jain metric is closer to 1, which indicates that the accuracy of the terrestrial devices is almost the same with the help of the global model. This indirectly reflects that using the reward function for selection after weighting can give all terrestrial devices an equal chance to participate in the aggregation process of the global model. Energy-oriented device selection scheme schedules users based on only a single criterion, energy consumption. This destroys the fairness of device scheduling as the number of training rounds increases. The scheme has the lowest Jain value, and this simulation result proves our point. In contrast, in the gossip scheme, there is randomness in the selection of devices for the UAV, and this randomness increases the fairness of the system. However, this scheme has too much randomness, thus causing the Jain metric curve to be too oscillating when converging.

### 5.4. Fairness Comparison under Different Parameters

**Figure 4:**
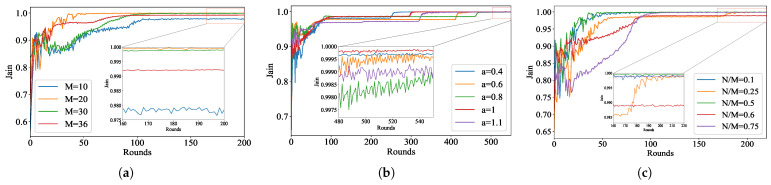
Comparison of Jain metrics with changing three parameters on MNIST dataset. (**a**) Changing the total number of devices. (**b**) Changing fairness parameter *a* in function. (**c**) Changing selection ratios with M=20.

We vary the total number of terrestrial devices, the size of the parameter *a* in the FM function, and the ratio of terrestrial device scheduling within this subsection with the aim of comparing the changes in system performance under different parameters. Firstly, we change the total number of terrestrial devices, and the specific results are shown in Figure 4a. It can be clearly observed that when the total number of terrestrial devices is 20, the Jain metric is the first to converge, and the convergence value is the largest. Therefore, we use M=20 when comparing with other algorithms, but as the total number of devices on the ground increases, the performance gradually deteriorates. When M=36, although the convergence rate is faster, the convergence value is lower. This illustrates the situation when an increasing number of terrestrial devices appear, which may require the assistance of multiple UAVs so that the resources can be allocated rationally.

Then, we change the size of the parameter *a* in the FM function and display the results, as shown in Figure 4b. From the global plot, we can clearly observe that the *a* value has little impact on the overall Jain value and more impact on the rate of convergence of Jain values. We zoom in on the local details and compare a=0.4 with a=1. Although the convergence is faster when a=0.4, its convergence value is smaller. Therefore, we use a=1 when making comparisons with other algorithms.

Finally, we change the ratio of device scheduling. We let the UAV select 2, 5, 10, 12, and 15 terrestrial devices to discuss the effect of different ratios on the fairness of the system, and the specific results are shown in Figure 4c. We can intuitively observe that the rate of convergence as well as the value of convergence is best when the ratio is 0.5. Therefore, we compare with other algorithms at this ratio. When the ratio exceeds 0.5, the more terrestrial devices the UAV selects to participate in the aggregation phase, the greater the degree of oscillation is for the first 100 rounds of FL. If considered in more urgent scenarios, the UAV cannot stay in a certain area for too long. At this point, as more terrestrial devices are involved in training, the overall system performance is worse in terms of fairness. Therefore, subsequent work can be done to investigate this further in scenarios with fewer global rounds.

## 6. Conclusions

The use of FL in emergency communication scenarios can improve the generalization of models while guaranteeing data privacy. UAVs can be widely utilized to assist communication in complex terrain due to their flexibility. However, due to the limited energy of terrestrial devices, it is not possible to upload data to UAVs in every round, and also the UAV cannot afford to receive data from all terrestrial devices. In this work, we consider the fairness issue of user scheduling and the long-term impact that the fairness issue brings to the disaster relief. We design a MAB algorithm for enhancing scheduling fairness, using the weighting function of model freshness and energy consumption as the reward function of the MAB problem, thus reducing energy consumption while ensuring efficient system performance. Simulation results demonstrate the advantages of this scheme in terms of identification accuracy, energy efficiency, and fairness compared to traditional schemes.

## Figures and Tables

**Table 1:** Experimental parameters.

Parameter	Value	Parameter	Value
Number of terrestrial devices	20	Radius of area covered	200 m
Energy budget	400 J	Transmit power of the UAV	1.258 W
Number of subchannels	10	Received power of the UAV	1.181 W
Bandwidth of subchannels	1 M	$\tau^{max}$	3 s
Transmit power of terrestrial devices	(0.1 W, 0.3 W)	*a*	1
Received power of terrestrial devices	(0.1 W, 0.3 W)	α	0.6

**Table 2:** The simulation results of algorithms.

Algorithm	Accuracy	Energy Consumption	Jain
Energy-oriented device selection	83.34%	231.62 J	0.9694
Our selection	91.06%	246.65 J	0.9995
Gossip selection	80.35%	373.19 J	0.9891

## Data Availability

No new data were created or analyzed in this study. Data sharing is not applicable to this article.
